# A de novo variant in *CASK* gene causing intellectual disability and brain hypoplasia: a case report and literature review

**DOI:** 10.1186/s13052-022-01248-z

**Published:** 2022-05-12

**Authors:** Ying Zhang, Yanyan Nie, Yu Mu, Jie Zheng, Xiaowei Xu, Fang Zhang, Jianbo Shu, Yang Liu

**Affiliations:** 1 Department of Neonatology, Tianjin Children’s Hospital (Tianjin University Children’s Hospital), No. 238 Longyan Road, Beichen District, Tianjin, 300134 China; 2 Graduate College of Tianjin Medical University, Tianjin, China; 3 Tianjin Key Laboratory of Birth Defects for Prevention and Treatment, Tianjin, China; 4 Tianjin Pediatric Research Institute, Tianjin Children’s Hospital (Tianjin University Children’s Hospital), No. 238 Longyan Road, Beichen District, Tianjin, 300134 China

**Keywords:** *CASK* gene, Gene variant, Intellectual disability, Brain dysplasia, Microcephaly

## Abstract

**Background:**

The pathogenic variation of *CASK* gene can cause *CASK* related mental disorders. The main clinical manifestations are microcephaly with pontine and cerebellar hypoplasia, X-linked mental disorders with or without nystagmus and FG syndrome. The main pathogenic mechanism is the loss of function of related protein caused by variant. We reported a Chinese male newborn with a de novo variant in *CASK* gene.

**Case presentation:**

We present an 18-day-old baby with growth retardation and brain hypoplasia. Whole-exome sequencing was performed, which detected a hemizygous missense variant c.764G > A of *CASK* gene. The variant changed the 255th amino acid from Arg to His. Software based bioinformatics analyses were conducted to infer its functional effect.

**Conclusions:**

In this paper, a de novo variant of *CASK* gene was reported. Moreover, a detailed description of all the cases described in the literature is reported. *CASK* variants cause a variety of clinical phenotypes. Its diagnosis is difficult due to the lack of typical clinical symptoms. Genetic testing should be performed as early as possible if this disease is suspected. This case provides an important reference for the diagnosis and treatment of future cases.

## Background

*CASK* gene located in Xp11.4 [1] and is an important gene in mammals, which plays a very important role in metabolic regulation and affects the development of postnatal brain [2]. *CASK* gene variants cause a wide range of human phenotypes. The pathogenic variants can lead to *CASK* related mental disorders. It is reported that *CASK* gene variant can mainly lead to these phenotypes: severe intellectual disability, microcephaly with pontine and cerebellar hypoplasia (MICPCH, OMIM: 300,749) in women. In men, mild to severe X-related mental disorders were observed with or without nystagmus, microcephaly and other malformations, and FG syndrome. The varied clinical phenotypes depend on the types of variants [3–5].

*CASK* gene encodes calcium/ calmodulin dependent serine protein kinase, which belongs to the membrane associated guanosine kinase (MAGUK) scaffold protein family. MAGUK protein plays an important role in the ionic channel targeting, anchoring and signal transduction of synapses, as well as regulating neural activity. *CASK* is a special member of p55 subfamily and is the only MAGUK which contains the calcium/ calmodulin dependent kinase (CaMK) domain at its N-terminal. *CASK* protein contains five domains, including two L27 (Lin2, lin7) domains, one PDZ domain and one integrated SH3 and GUK domain [6].

The *CASK* disorder is rare. ZHANG Yi et al. [7] reported a case of Chinese children in 2019 which is the first one in China. Here, we reported the second case and identified a de novo variant c.764G > A (p. Arg255His) of *CASK* gene in China. Bioinformatics software were used to predict the effects of the missense variant on the function of the *CASK* protein. Additionally, we reviewed the previously reported cases of *CASK* gene variants from different ethnic groups (Table 1), which contained the nucleotide changes, amino acid changes and clinical phenotypes caused by gene variants [8–23].

**Table 1:** Summary table of reported *CAS*K variants

Publication	No	Sex	Age	POP	LOC	Variant	AAC	TOV	Geno	Phenotype
Juliane Najm et.al. (2008) [21]	1	F	-	-	-	-	-	-	-	MICPCH, ID, deafness
	2	F	-	-	-	-	-	-	-	MICPCH, ID
	3	F	-	-	-	-	-	-	-	MICPCH, ID
	4	F	-	-	Ex21	c.1915C > T	p.(R639*)	Non	Hete	MICPCH, ID
	5	M	-	-	Ex9	c.915G > A	p. =	Spl	-	MICPCH, ID
Shin Hayashi, et al. (2008) [24]	6	F	5y	-	-	arrXp11.4p11.3 (41,500,243–45,480,187) × 1	-	-	-	MICPCH, DD, strabismus, nystagmus
Giulio Piluso et al. (2009) [3]	7	M	-	Ita	Ex2	c.83G > T	p. (R28L)	Mis	Hemi	FG, ID, hypotonia
Patrick S Tarpey et al. (2009) [25]	8	M	-	-	-	c.829C > T	p.(Y268H)	Mis	-	ID
	9	-	-	-	Ex22	c.2129A > G	p.(D710G)	Mis	-	ID, nystagmus
	10	-	-	-	-	c.2767C > T	p.(W914R)	Mis	-	ID, nystagmus
	11	-	-	-	-	c.1188C > T	p.(P396S)	Mis	-	ID
Anna Hackett et al. (2010) [4]	12	M	-	-	Ex22	c.2129A > G	p.(D710G)	Mis	-	ID, nystagmus, strabismus
	13	M	-	-	Ex27	c.2756 T > C	p.(W919R)	Mis	-	ID, nystagmus, epilepsy
	14	M	-	-	Ex8	c.802 T > C	p. (Y268H)	Mis	-	ID, epilepsy
	15	M	-	A-A	Ex13	c.1186C > T	p.(P396S)	Mis	-	ID, unsteady gait, resting tremor
	16	M	-	-	Ex23	c.2183A > G	p.(Y728C)	Mis	-	ID, cerebellar hypoplasia
	17	M	-	-	Ex26	c.2521-2A > T	p.(841_868 and p.841_843 del ALK)	Mis	-	ID, nystagmus
Ute Moog et al. (2011) [22]	18	F	10 m	Fre	In2	c.173-2A > C	-	Frs	-	ID, small nose, micrognathia,
	19	F	10 m	Fre	Ex3	c.174 T > A	p.(D58E)	-	-	ID, small nose, micrognathia,
	20	F	5y	Bri	In8	c.831 + 2 T > G	-	Frs	-	ID, epilepsy, BCH
	21	F	2y 10 m	Fre	In17	c.1668 + 1G > A	-	Spl	-	BCH, hypotonia, small nose
	22	F	4y	Ame	Ex5	c.379C > T	p.(E127*)	-	-	Axial hypotonia, peripheral hypertonia,
	23	F	8y	Ame	Ex17	c.1639C > T	p.(Q547*)	-	-	BCH, hypertonia, strabismus
	24	F	2y4m	Ame	In5	c.430–2 A > T	-	Spl	-	BCH, hypertonia, dyskinesia
Jun-ichi Takanashi et al. (2012) [26]	25	F	7y	Jap	-	c.173_173 + 1delGG	-	-	-	MICPCH, DD
	26	F	11y	Jap	-	c.2302 + 1 del T	-	-	-	MICPCH, DD
	27	F	8y	Jap	-	c.1910G > A	p.(G637D)	-	-	MICPCH, DD
	28	M	2y	Jap	-	c.1061 T > C	p.(L348P)	-	-	MICPCH, DD, epilepsy
	29	F	24y	Jap	Ex4	c.316C > G	p. (R106 *)	-	-	MICPCH, DD, epilepsy
Lydie Burglen et al. (2012) [27]	30	F	7y	-	Ex1-8	Xp11.4 deletion 0.3 Mb	-	-	-	PCH, ID, DD, FD, spasticity
	31	F	3y	-	Ex 1–27	Xp11.3-p11.4 deletion 3 Mb	-	-	-	ID, DD, deafness, FD, spasticity
	32	F	14y	-	Ex1	Xp11.4 deletion 0.5 Mb	-	-	-	ID, DD, FD spasticity
	33	F	13y	-	Ex21	c.1968G > A	p.(W656*)	Non	-	ID, DD, FD, epilepsy
	34	F	3y	-	In21	c.2040–2 A > G	-	Spl	-	ID, DD, deafness spasticity
	35	F	1y	-	Ex22	c.2080C > T	p.(Q694*)	Non	-	ID, DD, FD
	36	F	1y	-	Ex22	c.2074C > T	p.(Q692*)	Non	-	ID, DD, epilepsy
	37	F	10y	-	In24	c.2302 + 5G > A	-	Spl	-	ID, DD, deafness, epilepsy,
	38	F	14y	-	In21	c.2039 + 1G > T	-	Spl	-	ID, DD, FD, spasticity,
	39	F	8y	-	Ex21	c.1970G > A	p.(W657*)	Non	-	ID, DD, FD, stereotypies
	40	F	3y 6 m	-	Ex15	c.1501dupA	p.(M501fs)	Frs	-	ID, DD, FD, stereotypies
	41	M	15y	-	Ex4	c.[= /316C > T]	p.(R106*)mos	Non	-	ID, DD, FD, stereotypies
	42	M	13y	-	In3	c.278 + 1G > A	-	Spl	-	ID, DD, FD, epilepsy
Vassili Valayannopoulos,et al. (2012) [28]	43	F	13y	-	-	c.1970G > A	p.(W657*)	-	-	MICPCH, trunk ataxia, swallowing difficulties
	44	F	8y	-	Ex16	c.1577delG	p.(R526Sfs-X74)	Frs	-	MICPCH, trunk ataxia, dystonia, spasticity
	45		13y	-	Ex21	c.1968G > A	p.(W656*)	-	-	MICPCH, dystonia, swallowing difficulties
Hirotomo Saitsu, et al. (2012) [29]	46	M	4y	-	Ex2	(NG_016754.1: g.17883_129055del	deletion 111 Mb	-	-	MICPCH, OS, micropenis
	47	M	4y	-	Ex1	c.1A > G	p.(M1V)	-	Hemi	MICPCH, OS,
Shin Hayashi et al. (2012) [5]	48	F	2y 8 m	Jap	Ex2	c.79C > T	p.(R27*)	Non	-	ID, DD, deafness, microcephaly, hypotonia
	49	F	2y	Jap	-	c.316C > T	p.(R106*)	Non	-	ID., deafness, microcephaly, hypotonia
	50	F	2y 8 m	Jap	Ex27	c.2632C > T	p.(Q878*)	Non	-	ID, hyperopia microcephaly
	51	F	11 m	Jap	Ex3	c.243_244delTA	p.(Y81*)	Frs	-	microcephaly, ID
	52	F	7y 9 m	Jap	In4	c.357-1G > A	p.S119Rfs7X, p.H120Rfs22X	Spl	-	microcephaly, ID
	53	F	14y	Jap	-	c.2041-1G > C	p.W608Cfs29X, p.W608Cfs3X	Spl	-	Microcephaly, ID
	54	F	1y 9 m	Jap	-	arrXp11.4p11.3 (41,009,876–44,100,501) × 1	-	-	-	MICPCH, DD, deafness, hypotonia
	55	F	2y	Jap	-	arrXp11.4p11.3 (41,337,795–42,468,013) × 1	-	-	-	MICPCH, DD
	56	F	12y	Jap	-	arrXp11.4 (41,405,593–41,570,391) × 3	-	-	-	MICPCH, DD, epilepsy, strabismus
	57	F	2 m	Jap	-	arrXp11.4 (41,382,179–41,540,922) × 3 arrXp11.22 (56,012,908–56,275,153) × 3	-	-	-	MICPCH, DD, epilepsy, hypotonia, strabismus
Nakamura K. et al. (2014) [23]	58	M	-	-	Ex3	c.227_228del	p.(E76Vfs*6)	Frs	Hemi	PCH, TOF, epilepsy
JacquesL. Michaud et al. (2014) [30]	59	F	36 m	-	Ex2	c.82C > T	p.(R28*)	-	-	ID, cortical and cerebellar atrophy
Ute Moog et al. (2015) [31]	60	M	7 m	-	Ex7	c.704_708del	p.(K236Efs* 10ex7dn)	Frs	-	MICPCH, ID, DD, epilepsy, hypotonia
	61	M	10 m	-	-	dup ex10 – 16dn	-	-	-	MICPCH, ID, DD, epilepsy, hypotonia
	62	M	5y	-	-	c.1A > G ex1dn	-	-	-	MICPCH, ID, DD, epilepsy, hypotonia
	63	M	15 m	-	-	c.79C > T	p.(R27*ex2 dn)	-	-	MICPCH, ID, DD, epilepsy, hypotonia
	64	M	7 m	-	-	dup ex4–20 mos	-	-	-	MICPCH, ID, DD, epilepsy, hypertonia
	65	M	16 m	-	-	del ex1mos	-	-	-	MICPCH, ID, DD, hypertonia
	66	M	29 m	-	-	del ex3–9 mos	-	-	-	MICPCH, ID, DD
	67	M	20 m	-	-	dup ex1–5 mat	-	-	-	Microcephaly, DD
Tomoshi Nakajiri et al. (2015) [32]	68	F	13y	Jap	Ex21	c.1896dupC	p.(C633Lfs*2)	Frs	Hete	MICPCH, epilepsy
Patrick Rump et al. (2016) [33]	69	F	22y	-	-	c.2302 + 2 T > G	-	-	Hete	MICPCH, ID, DD, FD, epilepsy
Lucía Rivas et al. (2017) [34]	70	F	5y	-	-	deletion254.01 Kb	-	-	-	MICPCH, WS
Shin Hayashi et al. (2017) [35]	71	F	1y	-	-	c.868G > T	p.(E290*)	-	-	MICPCH, DD
	72	F	5 m	-	-	c.761-762delCT	p.(S246*)	-	-	MICPCH, hypotonia
	73	F	15y	-	-	c.1006-1012del ACCTCCT	p.(T336Qfs* 23)	-	-	MICPCH, epilepsy, DD, hypotonia
	74	F	4y2m	-	-	c.2103delT	p.(F710Lfs* 26)	-	-	MICPCH, DD
	75	F	1y	-	-	c.1677dupG	p.(R560Afs* 20)	-	-	MICPCH, DD
	76	F	17y	-	-	c.2508delT	p.(L837*)	-	-	MICPCH, DD, epilepsy
	77	F	11y	-	-	c.1896dupC	p.(C633Lfs*2)	-	-	MICPCH, epilepsy DD, hypertonia
	78	F	1y	-	-	c.1582 + G > A	-	-	-	MICPCH, DD
	79	F	3y	-	-	c.2302 + 1G > T	-	-	-	MICPCH
	80	M	4y 4 m	-	-	c.317G > C	p.(R106P)	-	-	MICPCH
	81	M	2y	-	-	c.[= /1493_1503 + 10delATGAACCAATGGTAAGTAGGAinsGG]	p.(D498Gfs* 12)	-	-	MICPCH, epilepsy DD, hypotonia
	82	F	6y4m	-	-	arrXp11.4p11.3 (41,618,898–43,755,475) × 1	-	-	-	MICPCH, DD, epilepsy
	83	F	4y	-	-	arrXp11.4p11.3 (41,145,925–46,090,321) × 1	-	-	-	MICPCH,
	84	F	12y8m	-	-	arrXp11.4p11.3 (41,163,139–44,592,980) × 1	-	-	-	MICPCH, DD, glaucoma, PHPV
	85	F	-	-	-	arrXp11.4 (41,442,660–41,527,850) × 3	-	-	-	Died
Bernt Popp et al. (2017) [36]	86	F	5y	Ger	Ex2	c.68del	p.(F23Sfs*18)	Frs	-	MICPCH, deafness, ID hypertonia
Stephanie C. DeLuca et al. (2017) [37]	87	F	54 m	-	-	c.2221 + 1G > C	-	-	-	MICPCH, single- word speech
	88	F	89 m	-	Ex17	c.1609C > T	p.(R537*)	-	-	MICPCH
	89	F	24 m	-	-	c.106C > T	p.(Q36*)	-	-	MICPCH, DD
P. Dunn, et al. (2017) [38]	90	M	6y 6 m	-	Ex26	c.2521–2 A > G	-	-	-	FG, nystagmus
Toshiyuki Seto et al. (2017) [39]	91	M	5y	-	Ex15	c.1424G > T	p.(S475I)	Mis	-	microcephaly, ASD
	92	F	3y	-	-	c.1424G > T	p.(S475I)	Mis	-	DD, ASD
Babylakshmi Muthusamy et al. (2017) [40]	93	M	14y & 17y	Ind	-	E550_dup	Stop gain and in-frame insertion	-	Hemi	microcephaly, clinodactyly
Xiuhua Bozarth et al. (2018) [41]	94	F	-	ME-C	-	c.2179–2181del GTA	p.(V727del)	-	Hete	infantile spasms, nystagmus, strabismus
Leslie E. W. LaConte et al.(2018) [42]	95	F	12y	-	-	c.1556 T > C	p.(M519T),	Mis	-	MICPCH, gait ataxia, nystagmus
	96	F	5y	-	-	c.1989G > A:	p.(G659D)	Mis	Hete	MICPCH strabismus
	97	F	9y	-	-	c.626 T > C	p.(L209P)	-	-	MICPCH, ID, motor disability
Hiroaki Murakami et al. (2019) [43]	98	F	5y	-	-	c.2041C > T	p.(R681*)	Non	-	microcephaly, BCH, DD, hypotonia
Francesca Cristofoli et al. (2019) [44]	99	F	25y	Eur	-	c.1315–7 A > G	p.(M438-A 439 insH*)	Spl	-	ID, DD, dystonia, small cerebellum
	100	F	21y	Eur	-	c.C109T	p.(Q37*)	Non	-	ID, DD, visual impairment
	101	F	6y	Eur	-	c.T626C	p.(L209P)	Nonsy-nonym-ous	-	ID, DD, FD, hypotonia, nystagmus
	102	F	17y	Eur	-	c.2302 + 1 G > A	p.(G741-H768 delinsD)	Spl	-	ID, DD, Visual impairment
ZHANG Yi, et al. (2019) [7]	103	M	3 m 27d	Chi	Ex20	c.1818_1821dup AACT	p.(T608Nfs* 16)	Frs	Hemi	MICPCH, DD, FD, hypertonia
This study	104	M	18d	Chi	Ex8	c.764G > A	p.(R255H)	Mis	Hemi	microcephaly, ID, DD, epilepsy, deafness

*Abbreviations*: *POP* Population, *LOC* Location, *AAC* Amino acid change, *TOV* Type of variant, *Geno* Genotype, *F* Female, *M* Male, *Ita* Italian, *A-A* African-American, *Fre* French, *Bri* British, *Ame* American, *Jap* Japanese, *Ger* German, *Ind* Indian, *MEC* Mixed-European Caucasian, *Eur* European, *Chi* Chinese, *Ex* Exon, *In* Intron, *Non* Nonsense, *Spl* Splicing, *Mis* Missense, *Frs* Frameshift, *Hete* Heterozygous, *Hemi* Hemizygote, *MICPCH* Microcephaly with pontine and cerebellar hypoplasia, *ID* Intellectual disability, *DD* Developmental delay, *FG* FG syndrome, *BCH* Brain stem and cerebellar hypoplasia, *PCH* Pontine and cerebellar hypoplasia, *FD* Feeding difficulties, *OS* Ohtahara syndrome, *TOF* Tetralogy of Fallot, *WS* West syndrome, *PHPV* Persistent hyperplasia of primary vitreous, *ASD* Autism spectrum disorder

## Case presentation

The patient was an 18-day-old male baby, gravida 3, para 1, born in full term with a birth weight of 2790 g. The condition of intrauterine distress was unknown, and the history of asphyxia was denied. Crying after birth but slightly weak. Apgar score was unknown. The couple denied the family genetic disease history. The patient was hospitalized in Tianjin Children's hospital mainly due to sucking weakness. When he was fed for the first time after birth, he was not willing to take the initiative to suck. He was fed with a spoon and could swallow. The infant rarely cries and cry weakly, with no fever and hoarseness, moaning and other symptoms. Admission examination: weight 2840 g, length 50 cm, head circumference 33 cm. The child's consciousness was weak and he occasionally had inspiratory laryngitis, hypotonia of the extremities. Holding reflex and embracing reflex were normal, the foraging reflex ( ±), sucking reflex ( ±). When he was crying, the corners of his mouth inclined to the left. His left nasolabial groove became slightly shallow along with right hand slightly hanging wrist, right foot slightly turned inward, and his right-hand pass-through palm. Laryngoscope showed that the arytenoid epiglottic folds on both sides were close to each other, and the mucosa was slightly tense. The cricoarytenoid joint were adducted, and the throat entrance was slightly blocked. Head Magnetic Resonance Imaging(MRI)showed that the bilateral frontal parietal lobes had slightly intense T1 and T2 signal shadows. The extracerebral space was widened, and the posterior angles of bilateral lateral ventricles were widened. Brainstem auditory evoked potential test showed deafness in the left ear and abnormality in the right brainstem. Ambulatory electroencephalogram examination and cerebrospinal fluid test were normal. Neuroelectrophysiological examination showed that there was no abnormality in facial nerve detection. After admission, the patient was given anti-infective treatment of Latamoxef disodium, expectorant therapy of ambroxol and other symptomatic treatment. Six days after hospitalization, there was no fever in the child and the supplementary feeding became better. The family members required to be discharged from the hospital.

Telephone follow-up at the age of 4–5 months, the symptoms of sucking weakness were slightly better than before. Later, spasm occurred at the age of 6 months and he was diagnosed epilepsy which was characterized by cyanosis of lips and clenching of both hands. After 9 months of oral medication with Sodium Valproate and Topiramate, there was no obvious improvement in condition. At present, the child was 14 months old, with a weight of 6000 g. He has microcephaly compared with children of the same age (family members did not measure the head circumference), accompanied by severe developmental delay and intellectual disability. He still can’t raise head, speak and walk.

**Fig. 1:**
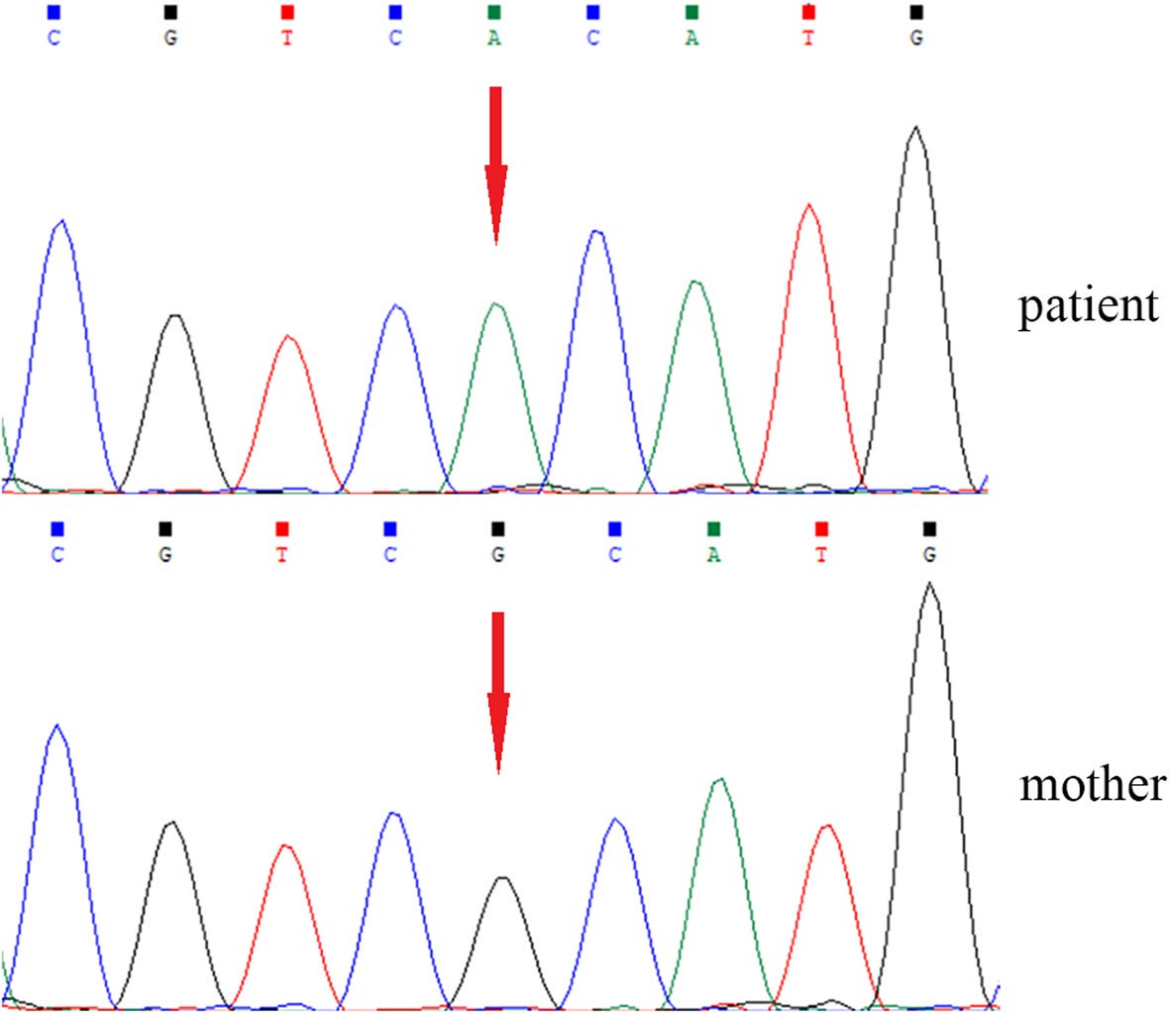
Sanger sequencing of a family with *CASK* gene c.764G > A missense variant

The results of Whole-exome sequencing (WES) showed that there was a hemizygous missense variant c.764G > A in exon 8 of *CASK* gene in proband. The variant changed the 255th amino acid from Arg to His. Because of the gene is located on the X chromosome, the paternal sample of the child does not need to be detected. Sanger sequencing of the child showed that the variant was not detected in his mother (Fig. 1). The pathogenicity classification of variants by American College of Medical Genetics (ACMG) guidelines [41] indicated that c.764G > A (p. Arg255His) is of pathogenic. The variant was not included in HGMD, 1000 Genomes, gnomAD and ESP6500 public databases.

**Fig. 2:**
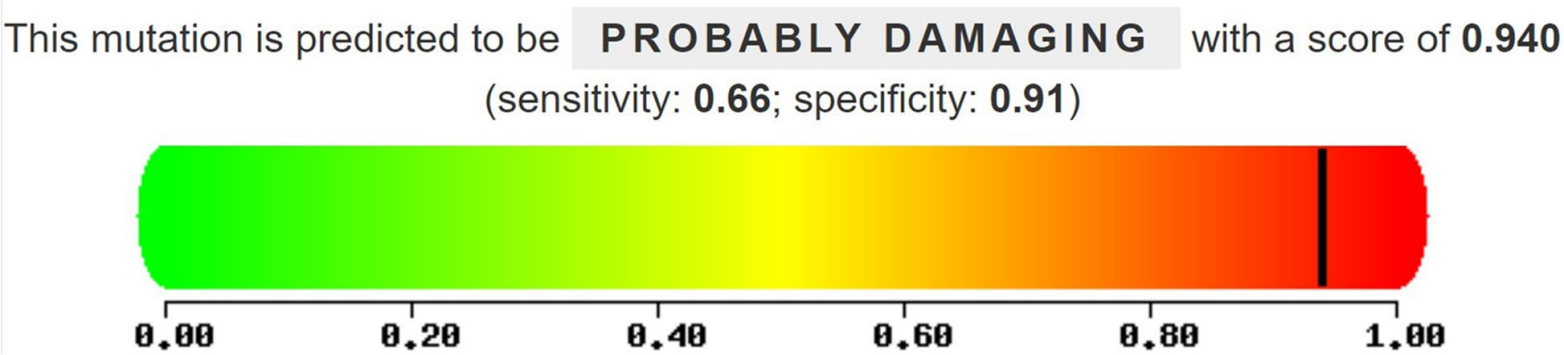
Conservation analysis of CASK protein sequences across different species. Amino acid positions of variants are highlighted in red box

**Fig. 3:**
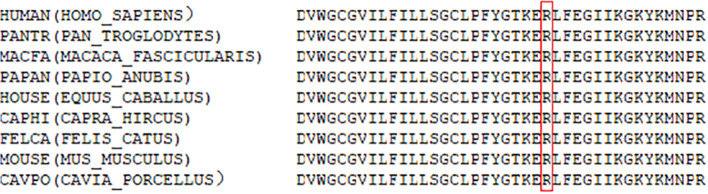
Prediction of functional effects of *CASK* variant

**Fig. 4:**
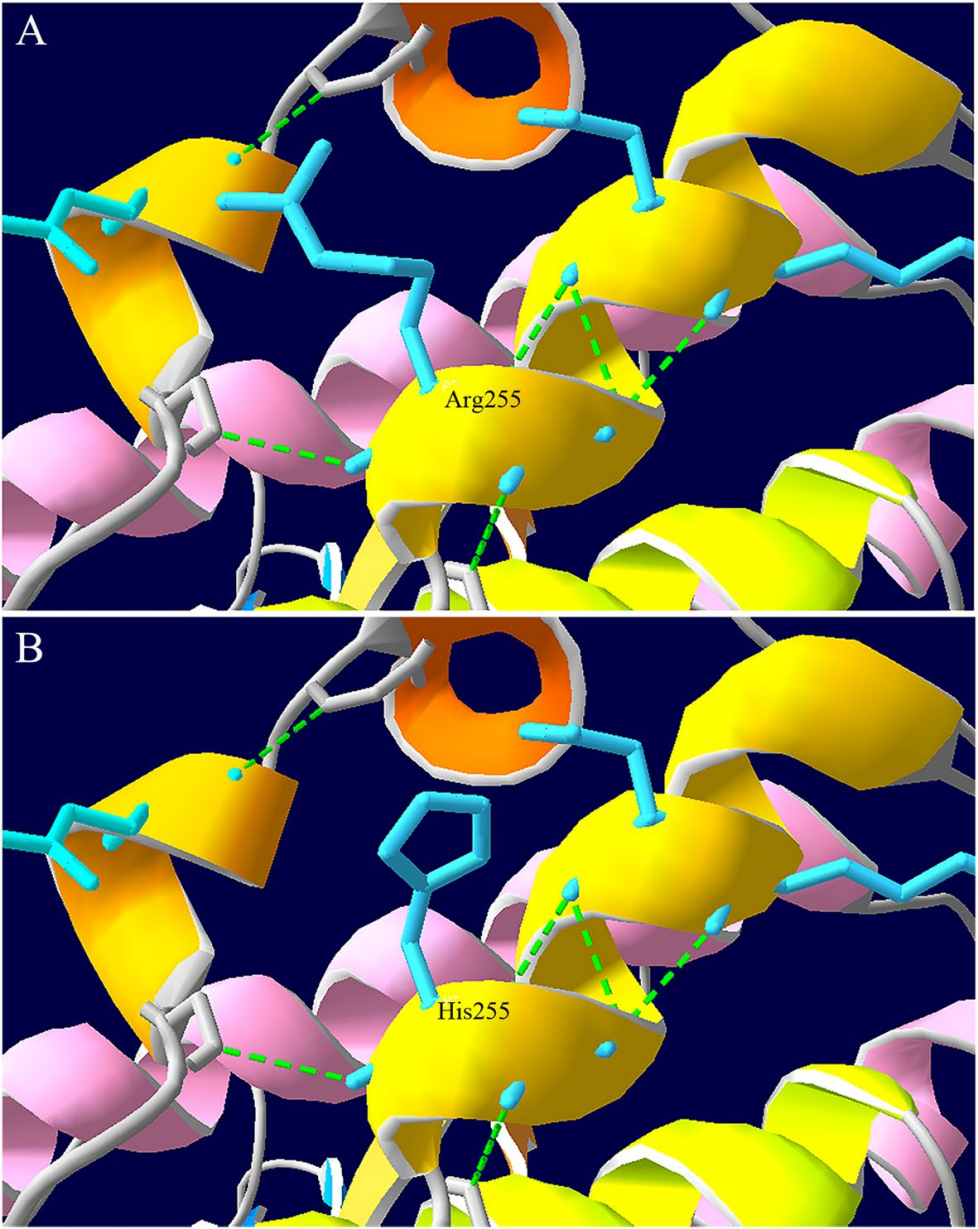
Three-dimensional structure model of CASK protein. Native(Arg, **A**)and mutant (His, **B**) side-chains at position 255 are shown in blue. The H-bonds are shown in dotted green line

Prediction of functional effects of *CASK* variant showed the c.764G > A variant was possibly damaging (Fig. 2). Amino acid sequence alignment showed that the variant occurred at a highly conserved residue in *CASK* with surrounding amino acid residues being conserved between orthologs (Fig. 3). Protein structure 3D modeling was performed. It was shown that the variant (p. Arg255His) had a damaging effect on the *CASK* protein structure stability (Fig. 4).

## Discussion and conclusions

*CASK* is widely distributed in different brain regions of mice. The insertion variant and targeted knockout of *CASK* gene cause the death of mice within 1–2 days after birth. The mice exhibit a cleft palate and apoptosis of thalamic cell increased. The research results indicate the important role of *CASK* gene in the nervous system [35]. In human fetal tissues, *CASK* is most expressed in brain, followed by kidney and lung, and the expression level of *CASK* in brain is 3–5 times higher than other organs [42].Although *CASK* is expressed in neurons, it is not limited to neurons. Studies have shown that *CASK* is widely present in basement membrane, lateral membrane or lateral basement membrane in different epithelial cells [45].

The structure of *CASK* suggests that *CASK* plays an important role in signal transduction, intercellular connection, cytoskeleton and binding to membrane proteins [33].*CASK* interacts with a variety of cell proteins and plays different roles according to the time and location of expression [37]. Firstly, it is involved in the formation of synapses and the interaction between synapses [46]. For example, *CASK* regulates axon growth and branch by interacting with Bcl11A [24]; Interaction between *CASK* and syndecan-2 regulates maturation of dendritic protein [25]. At presynaptic sites, *CASK* forms compound with MALS/Mint-1/Liprinα through its CaMK and L27A domains. This compound is involved in the organization of synaptic vesicles and regulates the release of neurotransmitters [26]. Secondly, *CASK* involves protein transport of NMDA glutamate receptor and synaptic target of N-type calcium channel. Through its PDZ and SH3 domains, *CASK* forms targeted interaction and regulation with neurexin-1 and ion channel synapses in a CDK5-dependent manner. Thirdly, *CASK* regulating gene expression and neurodevelopment. *CASK* can enter the nucleus and bind to a specific DNA sequence in the Tbr-1 complex. As a co-activator of Tbr-1, *CASK* induces the transcription of this sequence, so as to regulate the expression of genes related to the development of cerebral cortex, such as RELN [37]. Protein kinase A phosphorylation regulates the interaction between *CASK* and Tbr-1 and it is an important regulatory factor of *CASK* in the nucleus [27]. Y-P30 can control the nuclear localization of *CASK* in a cell adhesion molecule dependent manner [28]. *CASK* is involved in many cellular pathways, including mitochondrial, synaptic and protein metabolism. The dysfunction of these cells may be the basis of complex neurological diseases related to *CASK* dysfunction [29].

In 2008, Najm J et al. first reported the heterozygous deletion and variant of *CASK* gene in girls and boys with severe pontine and cerebellar hypoplasia [30]. Since then, 104 pathogenic variants of *CASK* gene have been identified through next generation sequence (Table 1). According to these publications, *CASK* variants cause a variety of clinical phenotypes. These cases shown that *CASK* gene does not have a hot variant site that causes pathogenic clinical phenotype. Inactivated variant is more common in female patients, and the clinical phenotype is more serious.

MICPCH is a rare X-linked disease, usually seen in women, characterized by neurodevelopmental delay, microcephaly, and pontocerebellar hypoplasia. The main clinical phenotypes of the disease are severe developmental delay or intellectual disability, microcephaly after birth, often accompanied by slow growth, language development disorders, axial muscle tone reduction with or without increased limb muscle tone, optic nerve hypoplasia and / or other eye abnormalities, such as nystagmus. Patients often have special facial phenotypes including microcephaly, protruding broad bridge and tip of nose, small nose or short nose, small jaw deformity, big ears, with varying degrees of pons and cerebellum hypoplasia and progressive aggravation, as well as hearing loss, epilepsy etc. [31, 32]. There are also some female patients without microcephaly and pontine dysplasia. Bozarth X et al. reported a case of early-onset infantile spasm caused by *CASK* frame deletion variant in a girl. Brain MRI showed focal supratentorial brain malformation. EEG showed peak rhythm disorder, but no MICPCH [34].

The relationship between genotype and phenotype of *CASK* variant is not clear. CASK inactivating variants appear to account for the majority of MICPCH cases and with severer phenotypes [36]. It is fatal to men in the prenatal or neonatal period. Najm J et al. reported a male child died at 2 weeks after birth. In addition to deletion or duplication variant, women with MICPCH phenotype also have heterozygous deletion variants, including nonsense, frameshift and splice site variants [30]. In general, *CASK* missense variant is common in boys with X-linked intellectual disability. The clinical phenotype is not very serious, and it is usually asymptomatic in girls. However, Laconte L E W et al. reported three women with *CASK* missense variant in heterozygote, and they have severe intellectual disability, microcephaly and hindbrain hypoplasia [38].

The child in our case was born with weakness sucking, decreased muscle tension of limbs, abnormal face, right hand and right foot deformity, deafness of left ear, epilepsy, microcephaly, serious developmental delay and mental disorder. The results of next generation sequencing showed that there was a hemizygote missense variant c.764gG > A, p. (Arg255His) in exon 8 of *CASK* gene in children. According to the classification of gene variation by ACMG, the variant could be classified as pathogenic. The patient was a male child with pathogenic missense variant. Compared with literature reports published, the missense variant is a de novo variant, and the clinical phenotype of the patient is consistent with the published cases.

MRI of *CASK* variant patients showed that the size of the corpus callosum was normal, the proportion of brain/ corpus callosum was low, and the area of brain, pons, midbrain, cerebellar vermis and hemispheres were reduced. Some studies have shown that MRI results of hypoplasia and normal or large corpus callosum in the middle and posterior brain of girls with microcephaly and neurodevelopmental delay should indicate the possibility of *CASK* variant, especially in the case of low brain / corpus callosum ratio [39].

In terms of disease diagnosis, WES is a powerful tool for the diagnosis of highly heterogeneous neurodevelopmental disorders [40]. Children with microcephaly will face lifelong psychomotor, cognitive and communication disorders. For this kind of children, their motor development is often delayed for several years, and they are far behind the children of the same age in intelligence and communication ability. These children usually have serious speech disorders. DeLuca SC et al. conducted intensive treatment on three girls with *CASK* gene heterozygote variant and MICPCH. Conducting targeted trials to improve fine and coarse motor skills, visual motor coordination, social and communication skills. Studies have shown that MICPCH children respond to intensive therapy aimed at improving function or independence [43]. The therapy can improve the life track and affect the quality of life. *CASK* is highly conserved in structure. LaConte LE et al. used a high-throughput imaging method to measure the misfolding tendency of *CASK* mutants, and proved that a chemical chaperone may be helpful to save the misfolding of *CASK* caused by missense variants. It providing a possibility for the treatment of structural variants in the future [44].

In summary, we reported a de novo variant of *CASK* gene. Moreover, a detailed description of all the cases described in the literature is reported. All published cases suggest that the variant of *CASK* can cause a variety of clinical phenotypes. Its diagnosis is difficult due to the lack of typical clinical symptoms. Genetic testing should be performed as early as possible if this disease is suspected. We believe that this case provides an important reference for the diagnosis and treatment of future cases.

## Data Availability

All data generated or analyzed during this study are included in this published article.

## Abbreviations

MICPCH: Microcephaly with pontine and cerebellar hypoplasia
MAGUK: The membrane associated guanosine kinase
CaMK: The calcium/ calmodulin dependent kinase
WES: Whole-exome sequencing
ACMG: American College of Medical Genetics
MRI: Magnetic Resonance Imaging
